# Stochastic modelling of membrane filtration

**DOI:** 10.1098/rspa.2016.0948

**Published:** 2017-04-26

**Authors:** A. U. Krupp, I. M. Griffiths, C. P. Please

**Affiliations:** Mathematical Institute, University of Oxford, Oxford OX2 6GG, UK

**Keywords:** fouling, filtration, porous-media flow, stochastic modelling

## Abstract

Membrane fouling during particle filtration occurs through a variety of mechanisms, including internal pore clogging by contaminants, coverage of pore entrances and deposition on the membrane surface. In this paper, we present an efficient method for modelling the behaviour of a filter, which accounts for different retention mechanisms, particle sizes and membrane geometries. The membrane is assumed to be composed of a series of, possibly interconnected, pores. The central feature is a *conductivity function*, which describes the blockage of each individual pore as particles arrive, which is coupled with a mechanism to account for the stochastic nature of the arrival times of particles at the pore. The result is a system of ordinary differential equations based on the pore-level interactions. We demonstrate how our model can accurately describe a wide range of filtration scenarios. Specifically, we consider a case where blocking via multiple mechanisms can occur simultaneously, which have previously required the study through individual models; the filtration of a combination of small and large particles by a track-etched membrane and particle separation using interconnected pore networks. The model is significantly faster than comparable stochastic simulations for small networks, enabling its use as a tool for efficient future simulations.

## 1. Introduction

Membrane-based processes are the method of choice for clarification, concentration, purification and separation tasks in industrial applications ranging from water desalination [1] to protein purification [2]. One of the key problems in any membrane-based process is fouling, which describes the blocking of pores by deposition of particles within or on the membrane. Fouling decreases the permeability of the membrane, thereby decreasing the efficiency of the process, with the membrane eventually needing to be cleaned or replaced.

Mathematical modelling plays an important role in membrane science, for example, in the area of understanding fouling, because the typical lengthscales range from a few to several hundred nanometres and hence direct observation of the filtration process becomes impractical. The retention and blocking mechanisms underlying fouling, therefore, have to be inferred indirectly from empirical quantities, such as the applied pressure difference or permeate flux, using mathematical models.

The standard model for membrane fouling describes the membrane as a plate of uniform depth with cylindrical holes and the particles as spheres of uniform size. It allows for four different blocking mechanisms, where the particles can (i) constrict the pores internally, (ii) seal the pores completely, (iii) seal the pores only partially or (iv) form an additional resistive layer, the *filtercake*; these mechanisms are termed *standard*, *complete*, *intermediate blocking* or *cake filtration,* respectively, and were developed by Hermans & Bredee [3] and Hermia [4].

While the four different blocking mechanisms have been derived by making rather simplistic assumptions about the pore geometry and the retention mechanisms, they are often sufficient to describe an entire filtration experiment. For example, Gironès *et al.* [5] modelled bovine serum albumin (BSA) filtration on silicon nitride microsieves using the complete-blocking law. De Lara & Benavente [6] observed cake filtration during BSA filtration with a polymeric membrane and standard blocking on a ceramic membrane, which is validated by scanning electron microscopy micrographs. Li *et al.* [7] reported best fits of cake filtration to the ultrafiltration of sodium alginate (SA) and intermediate blocking when subjecting SA to microfiltration. Finally, Bowen & Gan [8] found best fits of standard blocking to BSA separation, which is a common observation on the filtration of very dilute suspensions [9].

A single blocking mechanism is, however, rarely sufficient to model measurements such as the volume flux during a filtration experiment where the pressure difference across the membrane is kept constant. Iritani *et al.* [10] reported that a single blocking filtration law was insufficient to describe their experimental measurements for BSA filtration. Ho & Zydney [11] used a concurrent model of complete blocking and cake filtration to model protein fouling in direct stirred cell filtration, while Mondal & De [12] used a consecutive model for cross-flow filtration. Duclos-Orsello *et al.* [13] combined standard and intermediate blocking, as well as cake filtration, to obtain good fits to protein microfiltration.

Even the combination of the four blocking mechanisms, be it concurrent or consecutive, can at times be insufficient to model experimental observations appropriately. Ho & Zydney [14] combined intermediate blocking with a two-resistor model to explain different experimental results for straight-through versus interconnected membranes in protein microfiltration based on the membrane morphology. Bolton *et al.* [15] considered the special geometry of fibrous membranes to obtain better predictions for glass fibre filters.

The last two examples underline the need to allow for more complex geometries and pore–particle interactions in mathematical models for membrane fouling. In this regard, Dalwadi *et al.* [16] employed homogenization techniques to better understand the effect of porosity gradients in depth-filters, while Rahimi *et al.* [17] used full-scale finite-element simulations to model membrane filtration in more detail. A stochastic simulation approach was proposed by Griffiths *et al.* [18], who considered a three-step blocking process in a constant-pressure filtration setting. Here, in each pore, uniformly sized particles first constrict the pore by adhesion on the pore wall, then, once the radius of the pore has been sufficiently decreased, partially cover the pore and then finally stack up to form a filtercake above the pore. This blocking process is assumed to be the same for all pores, and so the flux through a given pore only depends on the number of particles it has retained. To compute the flux decline for the entire membrane they ran multiple stochastic simulations, where the probability of a pore receiving a particle in a given iteration step depended on the flux through it, the flux through the entire membrane was then obtained by summing over the fluxes of the single pores.

Stochastic models have been used to model particle transport and retention in porous media [19–21] and to provide an alternative approach to explain and model filtration. For example, Hsu & Fan [22] considered the number of blocked pores as a random variable and use the Carman–Kozeny equations to compute the corresponding pressure build-up in a deep porous medium. An alternative derivation of the complete-blocking model was proposed, which fits their experimental observations for filtering coal particles with a deep bed of sand. Iritani *et al.* [23] used a stochastic model to compute the expected number of retained particles in a filtration experiment. By considering the four blocking mechanisms used for standard, complete and partial blocking, and cake filtration, they obtain alternative derivations of the four blocking laws. Tarafdar *et al.* [24] modelled the filtration process as a transition between different *states* of the entire membrane, with each state corresponding to a certain flux through the membrane. In order to model depth filters, which primarily work by retaining particles within the membrane structure and not on the surface, Nassar *et al.* [25] subdivided depth filters in their model into several compartments to compute the spatial distribution of particles. For standard blocking of a deep porous medium by particles with a given, potentially continuous, size distribution, Shapiro *et al.* [26] derived a population balance model, which was then averaged to obtain a system of differential equations describing the reduction in permeability due to particle retention.

This paper builds on several of these ideas by introducing a generalization of the work of Griffiths *et al.* [18] to provide a tool to model membrane filtration for more complex particle–pore interactions. We focus on direct-flow filtration, where the particle-laden fluid flows orthogonally through the membrane. The fluid is pushed through the membrane by a constant applied pressure difference, and is commonly referred to as *constant-pressure filtration*. At the model’s core lies the assumption that the blocking process is the same for each pore. With this assumption, it follows that the *conductivity* of a pore, relating the applied pressure to the flux through the pore, only depends on the number of retained particles and, when there are different particle types involved, their order of arrival. We model the filtration as a stochastic process and consider the number of retained particles and their order for a given pore as our random variable. Taking an ensemble average over a large set of pores then allows us to derive a system of ODEs to compute the time-dependent probability distribution for our random variable.

Note that the number and order of arrival of the retained particles for a given pore effectively represents the state of a pore. Considering the state of a pore instead of the state of the entire membrane, however, has the advantage that we can determine the conductivity of a pore for a given state using analytical, numerical or experimental techniques. Moreover, it allows for insights into the randomness of the filtration as the distribution of the number of particles retained by the pores can be tracked during a simulation. The expected flux through the membrane can be obtained directly from the time-dependent probability distribution of the random variable and the state-dependent conductivity. Thus, this approach allows us to compute the decline in flux across the membrane for any set of particle–pore interactions where a relationship between the number of retained particles by a pore, including their order of arrival in case of different particle sizes, and the conductivity of the pore can be established.

Following the work of Griffiths *et al.* [27] on using stochastic simulations for interconnected membranes, we also show that this approach can be generalized to interconnected pore geometries. Instead of a compartmentalization as proposed by Nasser *et al.* [25], we represent the underlying membrane structure by a network of connected pores, extend the idea of retention-dependent conductivity to the links in the network and consider the change in pressure distribution through the network due to particle retention.

This paper is divided into three sections. In §2, we introduce our approach on the basis of monodispersed particles, which is extended to multi-particle models in §3. Finally, in §4, we discuss how to adapt our model to membranes with an interconnected pore structure.

## 2. Stochastic modelling of direct-flow filtration of monodispersed particles

### (a) A basic model of membrane filtration

**Figure 1.**
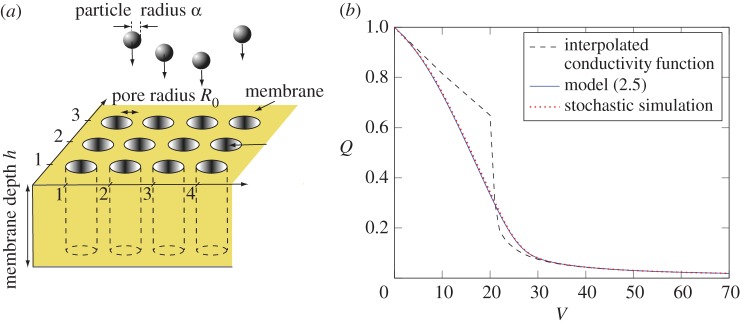
(*a*) Schematic diagram of a portion of the membrane set-up considered. (*b*) Flux (*Q*) versus throughput (*V*) graph for the stochastic simulation and ODE model (2.5) for the conductivity function (2.7), contrasted with the results for interpolated conductivity function. The stochastic simulation and our ODE model show excellent agreement, while the stark difference between the expected flux and the interpolated conductivity function underlines the difference between pore-level and membrane-level behaviours. (Online version in colour.)

We begin by considering the simplest membrane, comprising a solid material containing a number, *N*, of circular and non-interconnected holes called pores that run from one side of the membrane to the other. This type of structure describes, for example, track-etched membranes [28], where the pores are usually cylindrical or cone-shaped; a schematic is shown in figure 1*a*.

The particles in the fluid are assumed to be uniform in shape and size. Furthermore, we assume that the concentration *c* of the particles in the fluid is constant and that the particles do not interact. As the fluid passes through the pores of the membrane the particles are potentially retained with *retention probability* *P*_R_. The underlying mechanisms behind the retention are usually complex: they can be of physical or chemical nature, depend on properties of the particles and the membrane, and may change over the course of the filtration process.

We assume that the pores retain the particles in an identical way, that is, two pores having retained the same number of particles are assumed to be indistinguishable. Therefore, both the retention probability and the flux *f* through a given pore only depend on the number *n* of particles the pore has retained and the applied pressure difference Δ*p* across the membrane, that is, *P*_R_=*P*_R_(*n*,Δ*p*) and $f=f(n,\Delta p)=\tilde{\kappa }(n)\Delta p$, where $\tilde{\kappa }(n)$ denotes the *conductivity* of a pore with *n* retained particles. The last equality follows from the linearity of the Stokes equations, which describe the low-Reynolds-number flow encountered in membrane filtration of liquids. The conductivity function $\tilde{\kappa }$ will be a central concept in this work. This depends on several parameters, such as the geometry of the pores and the material properties of the membrane, and must be determined by analytical, computational or experimental considerations. The conductivity function is usually a decreasing function of *n*.

In typical constant-pressure filtration experiments, the flux $\tilde{Q}$ through the membrane is measured against the throughput $\tilde{V}$, the time-integral of the flux. For unit retention probability, we might anticipate that the flux through the entire membrane after processing a volume $\tilde{V}$ of fluid is simply the sum of the average flux through an individual pore after that time, i.e. $N\tilde{\kappa }(c\tilde{V}/N)\Delta p$, using linear interpolation whenever $c\tilde{V}/N$ takes a non-integer value. However, the measured flux $\tilde{Q}(\tilde{V})$ typically differs significantly from this expression. Hence the average behaviour of the flow through the pores, as seen in the flux, is not the behaviour of a pore with the average number of particles. The conductivity function $\tilde{\kappa }$ only describes the impact of particle retention on the flux through a single pore, whereas $\tilde{Q}(\tilde{V})$ combines this impact with the stochastic behaviour of the system composed of several pores. In the next section, we will therefore describe how to obtain the flux ($\tilde{Q}$) versus throughput ($\tilde{V}$) relationship from the conductivity function by accounting for the stochastic nature of the filtration process.

### (b) Derivation of evolution equations

From the assumptions of uniform concentration *c* of the particles in the fluid and no particle–particle interaction, it follows that the number of particles in a given volume $\Delta \tilde{V}$ of fluid is Poisson distributed with parameter $c\Delta \tilde{V}$. Hence, membrane filtration is fundamentally a stochastic process and the measured flux $\tilde{Q}$ against throughput $\tilde{V}$ constitutes an outcome of this stochastic process.

As the pressure difference Δ*p* across the membrane is uniform and the pores are non-interconnected, the flux through a given pore only depends on the number of retained particles and not on the flux through the other pores. Hence, the stochastic processes describing the filtration for the individual pores are independent of each other. Typical membranes have a large number of pores, and so the flux measurements will be close to the expected outcome of the stochastic process of the individual pores. This justifies framing filtration as a deterministic process and our goal is to determine the expected flux ${\tilde{Q}}_{e}$ as a function of time, from which we can derive the relationship as a function of volume.

For a given pore *i*∈{1,…,*N*}, we introduce the time-dependent random variable $X_{i}(\tilde{t})$, counting the number $n\in N_{0}$ of particles retained by the pore at time $\tilde{t}\geq 0$. We denote the probability of the pore having *n* particles at time $\tilde{t}$ by $P_{X}(n,\tilde{t}):=P(X(\tilde{t})=n)$. The measured flux ${\tilde{Q}}_{m}$ and the expected flux ${\tilde{Q}}_{e}$ are then given by

2.1 $$\begin{array}{rl} {\tilde{Q}}_{m}(\tilde{t}) & =\sum_{i=1}^{N}\tilde{\kappa }(X_{i}(\tilde{t}))\Delta p\\ \;\text{and}\;{\tilde{Q}}_{e}(\tilde{t}) & =\sum_{i=1}^{N}\sum_{n=0}^{\infty }\tilde{\kappa }(n)P_{X_{i}}(n,\tilde{t})\Delta p=N\sum_{n=0}^{\infty }\tilde{\kappa }(n)P_{X}(n,\tilde{t})\Delta p. \end{array}\}$$

To obtain the expected flux ${\tilde{Q}}_{e}$ we need to obtain the evolution equation for *P*_*X*_. We consider the change in *P*_*X*_ over a short time-period $\Delta \tilde{t}$, chosen to be small enough such that the probability of more than one particle arriving during this time is small. A pore will contain *n* particles at time $\tilde{t}+\Delta \tilde{t}$ if it contains *n* particles at time $\tilde{t}$ and either does not receive a particle during the interval $\Delta \tilde{t}$ or does not retain the particle it receives. This happens with probability

2.2 $$\begin{array}{rl} P & =P_{X}(n,\tilde{t})[\text{Poi}(0,\Delta \tilde{t}c\tilde{\kappa }(n)\Delta p)+(1-P_{R}(n))\;\text{Poi}(1,\Delta \tilde{t}c\tilde{\kappa }(n)\Delta p)+O(\Delta {\tilde{t}}^{2})]\\ & =P_{X}(n,\tilde{t})[1-P_{R}(n)\Delta tc\tilde{\kappa }(n)\Delta p\exp(-\Delta \tilde{t}c\tilde{\kappa }(n)\Delta p)+O(\Delta {\tilde{t}}^{2})], \end{array}$$

where we used the shorthand *P*_R_(*n*)=*P*_R_(*n*,Δ*p*) as the pressure difference is constant, Poi(*n*,λ) denotes the probability P(Y=n) where *Y* is Poisson distributed with parameter λ, and the *O*(Δ*t*^2^) correction accounts for the probability of retaining more than one particle in this time interval. A pore can also contain *n*≥1 particles at time $\tilde{t}+\Delta \tilde{t}$ by containing *n*−1 particles at time $\tilde{t}$ and retaining one particle during the time interval $\Delta \tilde{t}$. This happens with probability

2.3 $$\begin{array}{rl} P & =P_{X}(n-1,\tilde{t})P_{R}(n)\;\text{Poi}(1,\Delta \tilde{t}c\tilde{\kappa }(n-1)\Delta p)+O(\Delta {\tilde{t}}^{2})\\ & =P_{X}(n-1,\tilde{t})[P_{R}(n)\Delta \tilde{t}c\tilde{\kappa }(n-1)\Delta p\exp(-\Delta \tilde{t}c\tilde{\kappa }(n-1)\Delta p)]+O(\Delta {\tilde{t}}^{2}), \end{array}$$

where the $O(\Delta {\tilde{t}}^{2})$ correction accounts for the probability of retaining more than one particle during the time interval and having contained correspondingly fewer particles at time $\tilde{t}$.

Combining equations (2.2) and (2.3), letting $\Delta \tilde{t}\to 0$, scaling $\tilde{t}=(c/(\tilde{\kappa }(0)\Delta p))t$, so that on average initially one particle is expected to arrive in each pore per unit time step, $\tilde{Q}=\tilde{Q}(0)Q$, $\tilde{V}=(\tilde{Q}(0)c/N\tilde{\kappa }(0)\Delta p)V$ and $\tilde{\kappa }(n)=\tilde{\kappa }(0)\kappa (n)$, we obtain the dimensionless governing equations for the probability *P*_*X*_ as

2.4 $$\begin{array}{rl} \frac{dP_{X}(n,t)}{dt} & =-P_{R}(n)\kappa (n)P_{X}(n,t)+P_{R}(n-1)\kappa (n-1)P_{X}(n-1,t),\;n\geq 1\\ \;\text{and}\;\frac{dP_{X}(0,t)}{dt} & =-P_{R}(0)P_{X}(0,t), \end{array}\}$$

with initial conditions *P*_*X*_(0,0)=1 and *P*_*X*_(*n*,0)=0 ∀*n*≥1. Note that mass is conserved by summing the equations for *P*_*X*_(⋅,*t*) over all *n*.

As the flux is usually plotted against the throughput *V* , it is useful to rewrite (2.4) in terms of the expected throughput *V*_e_ by using d*V*_e_/d*t*=*Q*_e_, which gives

2.5 $$\begin{array}{rl} & \frac{dP_{X}(n,V_{e})}{dV_{e}}=\frac{1}{Q_{e}(V_{e})}[-P_{R}(n)\kappa (n)P_{X}(n,V_{e})\\ & \;\;\;+P_{R}(n-1)\kappa (n-1)P_{X}(n-1,V_{e})],\;n\geq 1\\ \;\text{and}\; & \frac{dP_{X}(0,V_{e})}{dV_{e}}=-\frac{1}{Q_{e}(V_{e})}P_{R}(0)P_{X}(0,V_{e}), \end{array}\}$$

with the same initial conditions *P*_*X*_(0,0)=1 and *P*_*X*_(*n*,0)=0 ∀*n*≥1 as for (2.4).

It is possible to obtain a closed-form solution for *P*_*X*_ for any *κ*. In the simplest case where *P*_R_*κ*=1 for all *n*≥0, we find that $P_{X}(n,t)=t^{n}/(n!)\exp(-t)$, the moments of the Poisson distribution with parameter 1, and that the flux *Q*_e_ is constant. In the case of a unit retention probability *P*_R_≡1 and a strictly decreasing conductivity function *κ*, that is 1=*κ*(0)>*κ*(1)>⋯ , which is expected to be the case in a filtration experiment, we obtain

2.6 $$Q_{e}(t)=\sum_{n=0}^{\infty }\kappa (n)P_{X}(n,t)=\sum_{n=0}^{\infty }\kappa (n)\left[\sum_{k=0}^{n}\left(\prod_{i=0,i\neq k}^{n}\frac{1}{\kappa (i)-\kappa (k)}\right)\exp(-\kappa (k)t)\right].$$

Other cases for the retention probability and conductivity function can be computed in a similar fashion using integrating factors.

It is important to note that, although we can compute an exact solution for an arbitrary retention probability *P*_R_ and conductivity function *κ*, the closed form is not particularly useful as the evaluation of the product in (2.6) is numerically unstable. Therefore, the numerical solution to (2.4) is the preferred method.

For simplicity, we have restricted ourselves in the derivation of equation (2.4) to a single-particle system with a one-dimensional state space, namely the number of retained particles by a pore. However, we can model more complex configurations that might occur within a pore, such as bridging, by introducing a multi-dimensional state space, where different states can correspond to different aggregations of the same number of particles within a pore or by subdividing the pore into several sections. The necessary techniques and generalizations for this are developed in §§3 and 4.

### (c) Comparison with a three-stage fouling model

Modelling filtration as a stochastic process is especially useful if there are different mechanical stages in the fouling of a pore. We compare our methods with the work of Griffiths *et al.* [18], who evaluate a three-stage fouling process using stochastic simulations on a fixed number of identical pores.

The three stages arise as the cylindrical pores have a larger radius than the spherical particles, allowing for the particles to enter the pores. Once a particle has entered a pore, it either adheres to the pore wall with retention probability *P*_R_ or passes through. The analysis we present holds for pore-radius- or time-dependent probabilities but here we shall assume that *P*_R_ is constant. It is assumed that the reduction in radius due to particle adhesion is uniform within the pore, so that a pore with initial radius *R*_0_ containing *n* particles has radius $R_{n}=\sqrt{R_{0}^{2}-n4a^{3}/3h}$, where *a* denotes the radius of the particles and *h* the depth of the pore; this is known as *standard blocking*. The new, reduced flux ${\tilde{Q}}_{n}$ through the pore can then be computed by using Poiseuille’s Law as ${\tilde{Q}}_{n}=\Delta pR_{n}^{4}/8\mu h$, where *μ* denotes the fluid viscosity.

As each retained particle decreases the radius of the pore, the maximum number of admissible particles per pore is given by *n*^⋆^=⌈3(1−*a*^2^)*h*/4*a*^3^⌉. Once the pore has a smaller radius than the particles due to this constriction, it enters the second stage, where the next particle that arrives at the pore will cover it, allowing for a fixed residual flux ${\tilde{Q}}_{\mathrm{res}}={\tilde{\kappa }}_{\mathrm{res}}\Delta p$. After the pore has been covered, subsequent particles reaching the pore will stack on top of each other in the third stage, forming a filtercake. The filtercake imposes an additional resistance that is proportional to its height.

Combining these three stages, we can infer the dimensionless conductivity function $\kappa =\tilde{\kappa }/\tilde{\kappa }(0)$ for the pore as

2.7 $$\begin{array}{r} \begin{array}{rl} \text{(first stage: constriction)}\;\kappa (n) & ={\left(1-n\frac{4a^{3}}{3\;hR_{0}^{2}}\right)}^{4},\;n\leq n^{⋆}\\ \text{(second stage: covering)}\;\kappa (n) & =\kappa_{\text{res}},\;n=n^{⋆}+1\\ \;\text{and}\;\text{(third stage: caking)}\;\kappa (n) & ={\left({\left(\kappa_{\mathrm{res}}\right)}^{-1}+r(n-n^{⋆})\right)}^{-1},\;n\geq n^{⋆}+2, \end{array}\} \end{array}$$

where the dimensionless parameter *r* relates the number of particles in the filtercake to its resistance.

To evaluate the flux–throughput behaviour of this model, in Griffiths *et al.* [18], *N*=144 pores were considered taking the average over several stochastic simulations using the following algorithm:

(i) Subdivide the throughput *V* into equal volume steps *V*_*k*_=*k*Δ*V* :=*k*/*c*, *k*=1,2,…, where *c* is the particle concentration (number of particles per unit volume), so that at each step a particle arrives at the membrane.
(ii) At each volume step *V*_*k*_, choose the pore to which the particle is assigned to, with the probability *P*_*j*_ of a given pore *j* receiving the particle being *P*_*j*_=*κ*(*n*_*j*_(*V*_*k*−1_))/*Q*(*V*_*k*−1_), where *n*_*j*_(*V*_*k*−1_) denotes the number of particles retained by pore *j* after volume *V*_*k*−1_ has been processed by the membrane.
(iii) If the particle, with probability *P*_R_(*n*_*j*_(*V*_*k*−1_)), is retained, update the number of retained particles of the pore that received the particle and continue.

To compute the expected flux *Q*_e_ for the conductivity function *κ* defined by (2.7), we first solve (2.5) where we extend the definition of *P*_R_(*n*) via *P*_R_(*n*)=1 if *n*≥*n*^⋆^ and *P*_R_(*n*)=0.1 otherwise, *r* was chosen to be 1, *a*=0.9, *h*=10, *R*_0_=1 and *κ*_res_=0.3. For a given volume *V* , we can then obtain *Q*_e_(*V*) using the same approach as in (2.1).

The solution to the system of ODEs (2.5) is shown in figure 1*b* to provide almost exact agreement with the stochastic model of Griffiths *et al.* [18]. The figure also crucially highlights the difference between the observed flux–throughput behaviour and the interpolated conductivity function that would be predicted if we assumed that the particles were equidistributed across the pores during the filtration. For a given non-zero retention probability *P*_R_, the expected dimensionless volume that is processed before a particle is retained is *P*^−1^_R_. Therefore, we choose interpolation points (*V*_*i*_,*κ*(*i*)) with $V_{i}=\sum_{k=0}^{i-1}P_{R}{\left(k\right)}^{-1}$ for plotting the conductivity function. This demonstrates why our model is essential to traverse from the microscopic level of the individual pore to the macroscopic level of the entire membrane. As the number of pores in a typical membrane is large, computing the expected flux *Q*_e_ is sufficient to predict the measured flux–throughput behaviour. Compared with the stochastic simulation, solving the evolution equation (2.5) offers a significantly faster and easy-to-implement method for this computation. In our simulations, we achieved a speed-up of three orders of magnitude and needed only a fraction of the lines of code.

### (d) Multiple-pore-geometry membranes

A multiple-pore-geometry membrane contains several types of differently shaped unconnected pores instead of a single pore type as assumed in the previous subsections. It is appropriate to consider these types of membranes because different pore geometries can be created in a single membrane either on purpose or due to imperfections in the manufacturing process.

For a membrane with *K* different types of pores, we can adapt the stochastic model to multiple-pore-geometry membranes by introducing representative random variables *X*_*k*_ counting the number of particles retained by pore type *k*∈{1,…,*K*}, *κ*_*k*_ denoting its conductivity function, *P*_*R*_*k*__ its retention probability and *P*_*X*_*k*__ the probability distribution of *X*_*k*_. As the flux through a given pore only depends on the number of particles retained by the pore, the time-dependent evolution equations for the different pore types are independent of each other. Hence, we can solve equation (2.4) for each pore type *k*∈{1,…,*K*} and then sum the different expected fluxes to obtain the total flux through the membrane. Specifically, if *N*_*k*_ denotes the number of pores of type *k*, we obtain the total expected dimensionless flux *Q*_e_ through the membrane as

2.8 $$Q_{e}(t)=\sum_{k=1}^{K}N_{k}\sum_{n=0}^{\infty }\kappa_{k}(n)P_{X_{k}}(n,t).$$

To obtain the volume-dependent evolution equation, we have to use (2.8) to compute the flux and take into account the number of pores of each type, hence

2.9 $$\begin{array}{rl} \frac{dP_{X_{k}}(n,t)}{dV_{e}} & =\frac{N_{k}}{Q_{e}(V_{e})}[-P_{R_{k}}(n)\kappa_{k}(n)P_{X_{k}}(n,t)\\ & \;+P_{R_{k}}(n-1)\kappa_{k}(n-1)P_{X_{k}}(n-1,t)],\;n\geq 1\\ \;\text{and}\;\frac{dP_{X_{k}}(0,t)}{dV_{e}} & =\frac{N_{k}}{Q_{e}(V_{e})}[-P_{R_{k}}(0)\kappa_{k}(0)P_{X_{k}}(0,t)], \end{array}\}$$

with initial conditions *P*_*X*_*k*__(0,0)=1, *P*_*X*_*k*__(*n*,0)=0 ∀*n*≥1 ∀*K*∈{1,…,*k*}.

We point out that the independence in the time evolution for the different pore types allows us to formulate the optimal pore-type composition of a membrane as an optimization problem of a linear objective function, however, the constraints are not necessarily linear. When designing a membrane, a standard goal is to maximize the expected throughput $V_{e}(T)=\int_{0}^{T}Q_{e}(t)\;dt$ of a membrane after time *T* in constant-pressure filtration by choosing the right mixture of pores *N*_1_,…,*N*_*k*_. Rearranging the expected throughput as

2.10 $$V_{e}(T)=\sum_{k=1}^{K}N_{k}\;\int_{0}^{T}\sum_{n=0}^{\infty }\kappa_{k}(n)P_{X_{k}}(n,t)\;dt=:\sum_{k=1}^{K}N_{k}V_{k}(T),$$

we see that obtaining a membrane with maximum expected throughput is in fact simply a packing problem, where we want to cover the surface of the membrane with the pore inlets such as to maximize (2.10). Packing problems are, in general hard problems, for which no efficient optimization algorithms exist. However, if the pore inlets have to be arranged on some lattice instead so that the total number of pores *N*=*N*_1_+⋯+*N*_*K*_ is prescribed, the maximum throughput will be attained by choosing only pores of type *k* where $V_{k}=\max\{V_{1},\ldots ,V_{K}\}$.

While this conclusion is as we would expect, we have highlighted the methodology that may be adopted to consider membranes with multiple pore geometries and that such generalizations are simple to include within our framework. In the remaining sections, we will return to single-pore-type models, but emphasize that all models can be extended to membranes with multiple pores using the results from this section.

## 3. Multi-particle systems

### (a) A two-particle system

We now turn our attention to the filtration of a fluid containing two types of particles: *small* particles, having a radius less than that of the pores, and *large* particles, having a radius bigger than that of the pores. We denote their concentrations by ${\tilde{c}}_{s}$ and ${\tilde{c}}_{l}$, respectively. As in the previous section, we consider a membrane of fixed depth *h* with cylindrical, non-interconnected pores of uniform radius *R*_0_ and apply a constant transmembrane pressure Δ*p*.

This two-particle model is a good approximation for filtering the protein BSA with sterile membranes having a pore diameter of around 200 nm. Protein monomers in general can aggregate to large polymers due to a variety of factors, even under operating conditions where the monomer state is highly favoured [29]. While BSA monomers have diameters of around 10 nm, the aggregated polymers can grow larger than the pore diameter. As an example, Ho & Zydney [14] measured particle size distributions between 100 and 450 nm for the BSA aggregates in a filtration experiment.

Having two types of particles extends the range of possibilities for a pore to foul. While small particles will enter the pores where, if retained with probability *P*_R_, they constrict the pore, particles that exceed the pore size will cover the pore entrance, allowing for a residual flux but making it impossible for any further small particles to enter the pore. Therefore, once a pore has been sealed, both small and large particles will stack up on the surface, forming a filtercake. For this model, we neglect the possibility of particles in the filtercake being released back into the fluid or filtercakes of neighbouring pores interacting with each other. The interaction between neighbouring filtercakes can be modelled by including pore–pore interactions as discussed in §4, whereas the release of particles back into the fluid would have to be dealt with by including additional terms in the governing equations (3.4) and (3.5).

Until the pore has been covered, we only have to keep track of how many small particles have been retained inside the pore. As soon as the pore is covered, we also have to keep track of the depth of the filtercake, that is we need to know how many small and large particles have been retained on the surface of the pore. We will identify the number of small particles *n* inside the pore and the number of large particles *l* and small particles *m* on the surface of the pore as the *state* of the pore; it is denoted by the triplet (*n*,*l*,*m*). A pore transitions from one state to another when it retains a particle. The newly attained state depends on the previous state as well as the particle that is being retained, we use the | sign to distinguish between two states that are attainable if either a small or a large particle is retained. As in §2c, we assume that there is a maximum number *n*^⋆^ of small particles that can be retained within a pore and every particle that arrives at the pore thereafter is retained on the surface. If no large particle has been retained yet, we therefore have to distinguish between these two cases:

3.1 $$\begin{array}{rl} \left(n,0,0\right) & ⟶(n+1,0,0)\;|\;(n,1,0),\;n<n^{⋆}\\ \;\text{and}\;(n^{⋆},0,m) & ⟶(n^{⋆},0,m+1)\;|\;(n^{⋆},1,m). \end{array}\}$$

Once a large particle has been retained, all particles are retained on the surface and so

3.2 (n,l,m) ⟶ (n,l,m+1) | (n,l+1,m),l≥1.

To obtain the conductivity function, we adapt the analysis from §2c, assuming that the radius of the pore shrinks in response to the deposition of particles so that we obtain an average pore constriction following the retention of each particle. In addition to the previous conductivity function (2.7), we introduce a further parameter *β*∈[0,1] to control the conductivity of a constricted pore. This is motivated by findings of Bowen & Gan [8], who observed a flux through fully constricted pores. Once the pore has been covered, all particles contribute to the formation of the filtercake which acts as an additional resistor in series, its resistance being proportional to the number of retained small and large particles. Hence, the total resistance of the pore is the resistance of the constricted pore and the resistance of the filtercake, and so the dimensionless conductivity is given by

3.3 $$\kappa (n,l,m)={\left({\left(1-\beta n\frac{4a^{3}}{3hR_{0}^{2}}\right)}^{-4}+l\;r_{l}+m\;r_{s}\right)}^{-1},\;n\leq n^{⋆},$$

where *a* denotes the radius of a small particle, *h* the depth of the membrane and *r*_l_,*r*_s_ relate the number of large and small particles, respectively, to the resistance of the filtercake. Reducing the parameter *β* increases the residual flux through a fully constricted pore.

To compute the probability density function for the random variable *X*, indicating the state (*n*,*l*,*m*) a given pore is in, note that the time-dependent evolution of the different pores is independent and the Poisson parameters for the transitions are given, as before (equation (2.2)), by ${\tilde{c}}_{s}\tilde{\kappa }(0)\kappa (X(t))\Delta p$ and ${\tilde{c}}_{l}\tilde{\kappa }(0)\kappa (X(t))\Delta p$, where $\tilde{\kappa }(0)$ denotes the dimensional conductivity of an unconstricted pore. Using (3.1), scaling $\tilde{t}=(({\tilde{c}}_{s}+{\tilde{c}}_{l})/(\kappa (0)\Delta p))t$, $c_{s}={\tilde{c}}_{s}/({\tilde{c}}_{s}+{\tilde{c}}_{l})$ and $c_{l}={\tilde{c}}_{l}/({\tilde{c}}_{s}+{\tilde{c}}_{l})$, so that initially one particle, either large or small, is expected to arrive per unit time step and *c*_s_ and *c*_l_ are the fractions of small and large particles, we obtain for (3.1)

3.4 $$\begin{array}{rl} \frac{dP_{X}((n,0,0),t)}{dt} & =-(c_{s}P_{R}(n)+c_{l})\kappa (n,0,0)P_{X}((n,0,0),t)\\ & \;+1_{n>0}c_{s}P_{R}(n-1)\kappa (n-1,0,0)P_{X}((n-1,0,0),t),\;n\leq n^{⋆}\\ \;\text{and}\;\frac{dP_{X}((n^{⋆},0,m),t)}{dt} & =-\kappa (n^{⋆},0,m)P_{X}((n^{⋆},0,m),t)\\ & \;+1_{m=0}c_{s}P_{R}(n^{⋆}-1)\kappa (n^{⋆}-1,0,0)P_{X}((n^{⋆}-1,0,0),t)\\ & \;+1_{m>0}c_{s}\kappa (n^{⋆},0,m-1)P_{X}((n^{⋆},0,m-1),t), \end{array}\}$$

where 1 denotes the indicator function taking value 1 if the associated condition is satisfied and 0 otherwise. Note that the retention probability *P*_R_ is only relevant for small particles while they can still enter the pore and so only depends on *n*. Similarly, for (3.2) we have

3.5 $$\begin{array}{rl} \frac{dP_{X}((n,l,m),t)}{dt} & =-\kappa (n,l,m)P_{X}((n,l,m),t)\\ & \;+c_{l}\kappa (n,l-1,m)P_{X}((n,l-1,m),t)\\ & \;+1_{m>0}c_{s}\kappa (n,l,m-1)P_{X}((n,l,m-1),t),\;l\geq 1. \end{array}$$

As before, we can obtain the expected dimensionless flux *Q*_e_ by summing over all possible states

3.6 $$Q_{e}(t)=\sum_{n=0}^{n^{⋆}}\;\sum_{l,m\in N}\kappa (n,l,m)P_{X}((n,l,m),t).$$

**Figure 2.**
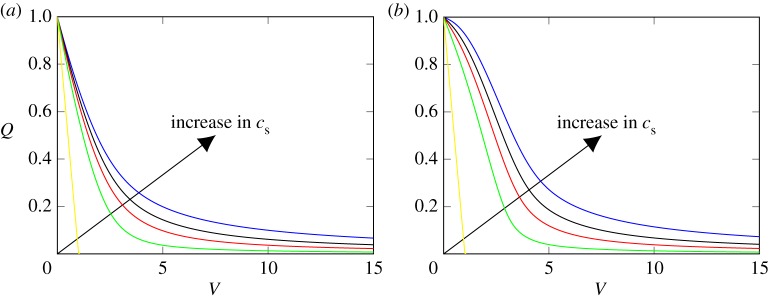
The effect of the residual flux parameter *β* and the ratio of large particles *c*_l_ on the flux (*Q*) versus throughput (*V*) relationship for the conductivity function (3.3) with parameters *a*=0.9, *h*=10, *R*_0_=1, *r*_s_=1, *r*_l_=10 and (*a*) *β*=1 and (*b*) *β*=0.1. The values for the ratio *c*_s_ are 0,0.8,0.9,0.95,1 and *P*_R_≡1. (Online version in colour.)

We consider the impact of the fraction of small particles *c*_s_ on the results of the membrane filtration based on the conductivity function (3.3) in figure 2. We contrast the effect of the residual flux in a completely constricted pore by considering the cases *β*=1,0.1.

### (b) General form of multi-particle models

For the general multi-particle problem, if we assume that there are *J* different types of particles then the state space can be represented by the set of ordered sequences of finite length, ${\left[J\right]}^{N_{0}}$. To derive the evolution equations for the probability *P*_*X*_ of the random variable *X*, we use the notation

3.7 $$\sigma \overset{\;c_{\sigma \tau }\;}{\to }\tau ,$$

denoting that a pore can transition from state *σ* to state *τ*, where $\sigma ,\tau \in {\left[J\right]}^{N_{0}}$, and that the relative concentration of the corresponding particles for the transition is *c*_*στ*_, their state-dependent retention probability is denoted by *P*_R_(*σ*,*τ*). The evolution equation for the probability *P*_*X*_ is then obtained by considering, for a given state *σ*, the probability of being in state *σ* and transitioning to *τ* and the probability of being in a state *γ* and transitioning to state *σ*, yielding

3.8 $$\begin{array}{rl} \frac{dP_{X}(\sigma ,t)}{dt} & =-\left(\sum_{\left\{\tau :\sigma \to \tau \right\}}c_{\sigma \tau }P_{R}(\sigma ,\tau )\right)\kappa (\sigma )P_{X}(\sigma ,t)\\ & \;+\left(\sum_{\left\{\gamma :\gamma \to \sigma \right\}}c_{\gamma \sigma }P_{R}(\gamma ,\sigma )\kappa (\gamma )P_{X}(\gamma ,t)\right). \end{array}$$

Note, however, that the set of ordered sequences of length at most *L* contains (1−*J*^*L*+1^)/(1−*J*)≥*J*^*L*^ elements; hence the state space for general problems will quickly become too large to be dealt with efficiently. If we cannot use state-space reduction schemes as described in §3a, Monte Carlo methods may be a viable alternative to obtain an approximation for the expected flux instead.

## 4. Modelling membranes with interconnected pore structure

### (a) General modelling strategy

Although the stochastic model developed in this paper is motivated by track-etched membranes with non-interconnected pores, it can also be applied to membranes with an interconnected pore structure, such as polyvinyl difluoride (PVDF) membranes [30]. In an interconnected pore structure, the inlets on the top surface of a membrane are connected to multiple outlets on the bottom surface and vice versa. In this case, there may be several paths through the membrane between connected inlets and outlets. Membranes with an interconnected pore structure exhibit a different flux–throughput behaviour to track-etched membranes in various filtration experiments [14].

**Figure 3.**
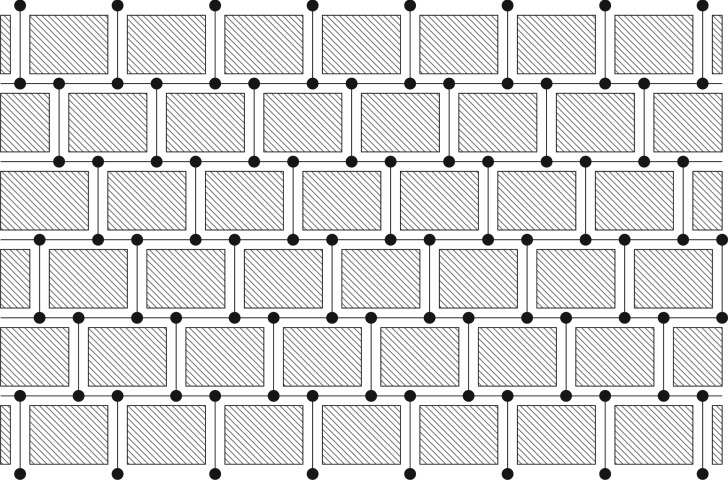
Representation of the internal membrane structure by superimposing an undirected graph onto the cross-section of a porous medium.

In order to evaluate the contribution of the membrane geometry to this differing behaviour, we represent the membrane structure beneath a given area, e.g. a square, by an undirected graph as shown in figure 3. The edges of the graph are identified with the pores discussed in the previous sections. However, they now no longer run all the way through the membrane but connect junctions instead, represented by the nodes in the graph. Accordingly, particles retained on the surface or within the membrane alter the conductivity of the corresponding edge and the state of an edge is given by the number and order of arrival of the particles the edge has retained. The flux through a given edge depends on the pressure above and below the pore and hence not only on the state of that edge but also on the states of the other edges in the network. We, therefore, introduce the state of the network as the Cartesian product of the states of the edges in the network.

We assume that the expected time for retaining a particle within the network is much smaller than the expected time between two particles arriving, and so the particle arriving in the structure is retained before the next particle arrives. Thus, the network transitions from one state *σ* to another state *τ* if the two states differ by an edge *e* having retained one more particle in *τ* than in *σ*, which we denote by $\sigma \overset{e}{\to }\tau$. The derivation of the evolution equation for the probability distribution of the state of the network, similar to (2.4) and (3.8), is divided into two steps. For a given state *σ* of the network, we first compute the pressure distribution within the network. Then, for each edge *e*, we compute the probability for *e* to retain the next particle.

As for the pores in the previous sections, the state-dependent conductivity function for a given edge can be computed either analytically or numerically, or determined experimentally. The pressure distribution in the network of state *σ* can then be obtained by solving the corresponding set of linear equations that arise due to conservation of mass: for a given node *v*, let *p*_*v*_ denote the pressure at the corresponding junction, for an edge *e*=*e*(*u*,*v*)=*e*(*v*,*u*) connecting vertices *u*,*v*, let *κ*(*e*,*σ*)=*κ*(*e*(*u*,*v*),*σ*) denote its conductivity. If the node is located at the upper or lower surface of the membrane, we have *p*_*v*_=*p*_in_ or *p*_*v*_=*p*_out_ as boundary conditions, as we assume that the inlet and outlet pressures are constant. Within the network, we have conservation of mass, implying for every node *v*

4.1 $$\sum_{u\in C(v)}\kappa (e(v,u),\sigma )(p_{v}-p_{u})=0,$$

where *C*(*v*) denotes the set of nodes *u* that *v* is connected to. In order to obtain a unique solution to the resulting set of linear equations, we only consider those vertices that can be reached from either one of the inlet or outlet nodes via a path of edges having non-zero conductivity.

In the previous models, the concentration of particles arriving at a pore was known and constant. However, in a network the concentration of particles in the fluid leaving a pore may change as particles are being retained. As a consequence, the Poisson parameter *Λ*(*σ*,*τ*) for the transition $\sigma \overset{e}{\to }\tau$ between two states *σ*,*τ* is no longer given by the product of the concentration *c*_*στ*_, the flux, and the retention probability *P*_R_(*e*,*σ*,Δ*p*(*e*,*σ*)) alone, with Δ*p*(*e*,*σ*) denoting the (positive) pressure difference across *e*. We now also have to factor in the probability of a particle reaching the edge *e* in the first place. For a particle to reach the edge *e*, it has to traverse the network along a path ending in *e* without being retained. Since the particles travel with the fluid, the pressures in the nodes of the path have to be decreasing and so there is only a finite number of these paths. At every node *v*, since there can be multiple edges to travel along, we assume that the probability *P*_*J*_(*l*,*σ*) of taking an edge *l* leading to a node *u* with lower pressure is given by the flux through *l* divided by the sum of the fluxes of the edges leading from *v* to nodes with lower pressure. Thus, we obtain the Poisson parameter

4.2 $$\begin{array}{rl} \Lambda (\sigma ,\tau ) & =c_{\sigma \tau }\;\kappa (e,\sigma )\Delta p(e,\sigma )\\ & \;\cdot \left[P_{R}(e,\sigma ,\Delta p(e,\sigma ))\sum_{w\in \text{Paths}(e)}\prod_{l\in w\setminus e}P_{J}(l,\sigma )[1-P_{R}(l,\sigma ,\Delta p(l,\sigma ))]\right], \end{array}$$

where Paths denotes the set of all paths with decreasing pressure on the nodes that end with *e*. Note that the Poisson parameter defined in (4.2) is independent of time and only depends on the state of the network.

Similar to equation (3.8), we obtain

4.3 $$\frac{dP_{X}(\sigma ,t)}{dt}=-\left(\sum_{\left\{\tau :\sigma \to \tau \right\}}\Lambda (\sigma ,\tau )\right)P_{X}(\sigma ,t)+\left(\sum_{\left\{\gamma :\gamma \to \sigma \right\}}\Lambda (\gamma ,\sigma )P_{X}(\gamma ,t)\right).$$

As the state space of the network is given by the Cartesian product of the state spaces of the different edges, solving the system of ODEs arising from (4.3) quickly becomes infeasible. We therefore either have to look for symmetries or other simplifications to obtain a state space of manageable size (see §4b) or consider small networks (see §4c). If neither is an option, stochastic simulations, as presented by Griffiths *et al.* [27] are more suitable to compute a good approximation for the expected flux through the network.

### (b) Example: retention of large particles in two- and three-dimensional grids

We begin by considering a simple model for the retention of particles with larger diameter than the pores, so that all particles in the fluid are retained on the surface. We assume that a particle landing on an inlet seals it completely. As the particles cannot enter the membrane due to their size, only the edges incident to an inlet can change their conductivity due to particle retention. Hence, the state space of the network can be simplified to {sealed,unsealed}^*N*^, where *N* denotes the number of inlets, i.e. pores connected to the upper surface (generalizing our previous definition for the number of pores in a non-interconnected membrane).

**Figure 4.**
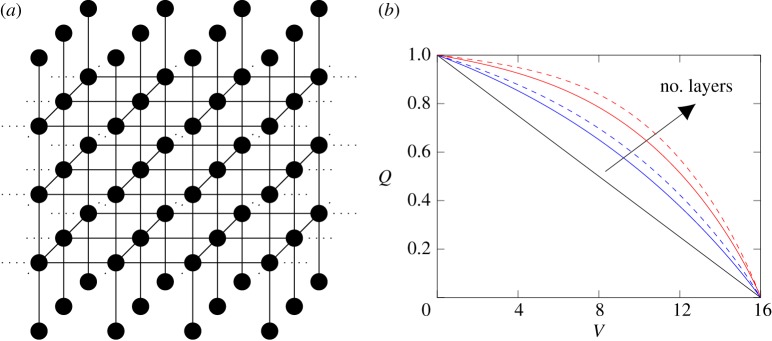
(*a*) Schematic of a 3×4 three-dimensional grid structure with five layers. The dotted lines illustrate the cyclic connection. (*b*) Flux (*Q*) versus throughput (*V*) graphs for the 4×4 (dashed) and 16×1 (solid) configuration with four (blue) and eight (red) layers.The black line corresponds to 16 non-interconnected pores. An increase in concavity is observed with an increase in the number of layers, with the concavity for the three-dimensional structure being more pronounced than for the two-dimensional structure with the same number of layers. (Online version in colour.)

To evaluate the impact of the underlying membrane structure, we consider a simple network as shown in figure 4*a*, where we are interested in the effect of the depth of the network on the decline of the expected flux. Every edge in the network has initially unit dimensionless conductivity. If one of the nodes of the edge is an inlet, the conductivity is reduced to zero once the inlet is sealed.

While the network of a real interconnected membrane is usually three-dimensional, it is useful to consider a two-dimensional model. We show the difference in the flux–throughput graphs for the two- and three-dimensional structure by comparing the results for a 4×4 and 16×1 gridstructure, and a membrane containing 16 non-interconnected pores. To provide the most accurate representation of a real membrane, we introduce cyclic connectivity, meaning that the nodes on the right boundary are connected to the ones on the left and the ones on the front boundary to those at the back.

Compared with a membrane with non-interconnected pores, the flux–throughput graph increases in concavity with the number of layers of the membrane (figure 4*b*), a pattern that is commonly observed when filtering BSA with interconnected membranes. Additionally, the concavity of the flux–throughput graphs is more pronounced for the three-dimensional structure than the two-dimensional structure, which we expect to be due to the higher average degree of the vertices in the three-dimensional structure (figure 4*b*).

### (c) Internal blocking in a small network

**Figure 5.**
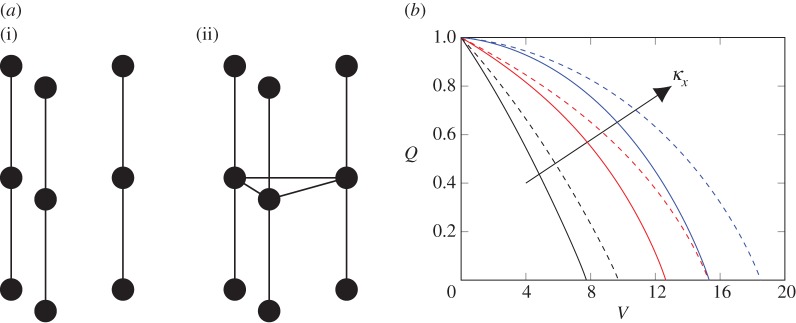
(*a*) Schematic of non-interconnected (i) and interconnected (ii) network structure. (*b*) Flux (*Q*) versus throughput (*V*) graph for *κ*_*x*_=0.1, 0.5, 0.9 for interconnected (solid) and non-interconnected (dashed) network structure. The interconnected network structure leads to a larger expected total throughput than the non-interconnected structure for the chosen retention probabilities. (Online version in colour.)

To study the impact of particle size on the flux–throughput shape for different membrane geometries, we now consider a model of a single particle type with the pores having larger diameter than the particles. In this case, initially the particles can traverse any edge in the graph. We assume that the dimensionless conductivity of an edge is either 1,*κ*_*x*_ or 0 depending on whether the edge has retained 0,1 or 2 particles, respectively, with differences in the value of *κ*_*x*_ identified with differences in the particle size. We consider *κ*_*x*_=0.1,0.5,0.9 where a smaller particle size corresponds to a bigger *κ*_*x*_. As in §2c, we choose a retention probability *P*_R_=0.1. Thus, the state space of an edge can be represented by the set {0,1,2} and the size of the state space is 3^*E*^, where *E* is the number of edges in the network. We consider the two networks shown in figure 5*a* with six and nine edges for the non-interconnected and interconnected networks, respectively. The state space for the interconnected network with nine edges contains 3^9^=19 683 elements, which makes computing the expected flux for the interconnected network computationally still feasible. However, considering more than 10^5^ states becomes difficult without further optimization.

Note that for the same values of *κ*_*x*_, the flux–throughput curve for the interconnected network structure lies above the flux–throughput curve for the non-interconnected structure, and the expected total throughput is significantly higher for the interconnected network structure than for the non-interconnected structure (figure 5*b*). This is an observation that we expect in general when comparing the performance of interconnected and non-interconnected membranes, and is corroborated by the stochastic study performed in [27], as an interconnected membrane structure allows for more load balancing once the pores start to constrict.

## 5. Conclusion

In this paper, we have shown how to compute the expected flux through a membrane during a filtration experiment on the basis of a *conductivity function*, which relates the number and order of the retained particles in a pore to the flux through it. The expected flux through the membrane can then be computed by solving a system of ODEs for the probability distribution of the state of a given pore. Describing membrane filtration in terms of a conductivity function opens up the possibility of considering a variety of retention mechanisms in filtration models, as the stochastic model is valid for any given non-negative conductivity function. We show that this approach can be applied to a range of membrane-type to feed-type combinations by discussing its application to multi-particle feeds, multi-pore-type membranes and membranes with an interconnected pore structure. Furthermore, for typical applications, the size of the state space is such that the computation of the expected flux is orders of magnitude faster and more precise than using a stochastic simulation. Finally, the derivation of the ODEs highlights that membrane filtration fundamentally is a stochastic process and the ODE model allows us to compute the probability distribution of the potential states of a pore.

Our model is based on the assumption that one particle enters a pore or interconnected pore structure at a time and so we can define a conductivity function relating the number of retained particles to the flux through the pore. For high enough particle concentrations, several particles can be expected to traverse a pore at the same time and so the assumptions underlying our derivations are no longer valid, making our method not applicable for these instances. When dealing with general multi-particle models or intricate geometries, additional considerations or assumptions have to be made to reduce the size of the state spaces for the corresponding system of ODEs, otherwise the method becomes already impractical for modest instances. If these reductions in the state space are not possible, computing an approximation for the expected flux should be considered instead.

In the realm of interconnected membranes, future work can include deterministic–stochastic models, where certain retention processes are assumed to follow a deterministic, easy-to-evaluate law, whereas others are assumed to be stochastic. This approach would allow us to consider, for example, several-particle-type feeds through interconnected membranes. Based on the ansatz of modelling fouling as a stochastic process, it would furthermore be important to find ways of computing the probability density for the measured flux. These insights could be of significant value for the area of microfluidic filtration, where the small number of particles and pores often shows the stochastic nature of the filtration process.

## Supplementary Material

Numerical_Code

## Data accessibility

The numerical implementations can be found in the electronic supplementary material.
